# The combined effect of patient classification systems and availability of resources can bias the judgments of treatment effectiveness

**DOI:** 10.1038/s41598-025-01043-w

**Published:** 2025-05-07

**Authors:** Aranzazu Vinas, Fernando Blanco, Helena Matute

**Affiliations:** 1 Department of Psychology, Deusto University, Bilbao, Spain; 2 Department of Social Psychology, University of Granada, Granada, Spain; 3 Mind, Brain and Behavior Research Center (CIMCyC), Granada, Spain

**Keywords:** Scarcity, Patient classification systems, Causal illusion, Decision-making, Cognitive bias, Causal judgments, Psychology, Health care

## Abstract

Patient classification systems (PCS) support clinical decision-making but may rely on incorrect, outdated, or insufficient data. Doctors can sometimes override errors using their experience. However, certain factors such as scarcity of resources could lead to reliance on incorrect PCS recommendations, with consequences for patients. We conducted two experiments where participants interacted with a PCS that incorrectly classified fictitious patients as more or less sensitive to a treatment. Participants had the opportunity to administer the treatment on a series of patients, and use the feedback to learn that the PCS was wrong and all patients were equally sensitive. This was tested in contexts of abundant and scarce resources. Additionally, the treatment was effective in Experiment 1, but ineffective in Experiment 2. Results indicate that people generally trust the PCS recommendation, to some extent neglecting the information they collect during the task. This can lead to uneven resource allocation, especially in scarcity conditions, and incorrect perceptions of effectiveness, which in Experiment 2 implies believing that an ineffective treatment works. We preregistered the experiments, and all data and materials are public.

**Supplementary Information:**

The online version contains supplementary material available at 10.1038/s41598-025-01043-w.

In clinical practice, patients are often classified to allocate the resources efficiently, targeting those with greater needs, or those with higher chances of treatment success. This approach is commonplace when defining vaccination programs and screening protocols for early detection of diseases such as cancer. Specifically, in these situations, the target population is usually determined based on scientific evidence about incidence, alongside other criteria, and all while weighting economic factors. For example, the European Commission’s most recent recommendation for breast cancer is to define national screening protocols directed to women aged between 45 and 75^1^, but different countries then define different protocols as a function of their resources and other variables. For example, the Spanish Ministry of Health’s recommendation is to define protocols for breast cancer screening directed to women between 50 and 69^2^.

Moreover, categorizing patients is also a common practice at the beginning of the treatment processes. Patient Classification Systems (PCS) are decision-making support systems, increasingly used in healthcare^3,4^, which intend to measure and predict the necessary resources for each person^5^. Examples of widely used PCSs are (1) The Manchester Triage System, which categorizes patients based on urgency levels (non-urgent, standard, urgent, very-urgent, immediate) and is adopted worldwide^6^; and (2) Diagnosis Related Groups (DRG), introduced in the early 1980s and widely used in Europe, the United States, Latin America, and Australia^7,8^. The DRG was designed to reduce hospital administration costs and has proved to effectively do it, although it is still unclear whether it has succeeded in improving healthcare quality^8^. PCSs like the Manchester Triage System, the DRG, and also more recent ones based on artificial intelligences, typically rely on medical criteria (e.g., abdominal pain, overdose), but they also include previous medical history and demographic data such as gender or age (for example, Busse et al., 2013)^7^.

Additionally, healthcare professionals can treat patients differently without using predefined protocols, often due to unconscious, unintended, and implicitly biased decisions^9–11^. In this respect, the previous evidence shows that specific groups of patients are systematically and negatively discriminated against according to different factors, such as previous disabilities^10,12^, gender^10^, ethnicity^9,10,13,14^, and age^15^. Sometimes even the left digit of their age (e.g. 70 vs. 69 or 60 vs. 59) can be a factor that influences the doctors’ unconscious classifications and decisions^16^.

Unfair allocation of healthcare resources became particularly evident during the COVID-19 pandemic when some argued that due to the scarcity of health resources, these had to be allocated efficiently, often favoring a specific demographic group, under the assumption that the scarce resources available would be more effective for them as compared to other groups^17^. This favored group usually comprised young, healthy individuals with no previous pathologies. To provide some examples worldwide: an analysis on resource allocation criteria during the pandemic in the US showed a shift from patient-centered to community-centered guidelines, thus limiting the access that patients with disabilities had to critical resources (treatments, ICU beds, etc.)^17^; in Spain, patients living in nursing homes were also deprived from treatments^18^; in South Africa and probably in other countries with limited resources, access to vaccines was prioritized so that older people were effectively discriminated^19^. Although we cannot confirm this in all cases, many of these classifications were probably incorrect in the sense that they were not properly justified by a clinical analysis, and therefore caused evident harm to certain groups that has even been taken to court. For example, in Spain, some associations of relatives of people who died in nursing homes have coordinated to file complaints against regional health officials during the pandemic^20^. Thus, it is interesting to gain insight into how the decisions people make in the medical context can be altered by the combination of scarcity and PCS.

Moreover, the above observations are, in fact, consistent with previous research showing that scarcity influences our judgments and decisions, with consequences in different domains, including health^21–26^. Notably, in a recent experimental series, Vinas et al.^27^ found that scarcity (operationalized as a limited budget to treat patients) led participants to reduce their use of a fictitious pseudomedicine in a fictitious scenario, in which they played the role of doctors. Pseudomedicines are treatments presented as effective but lacking scientific evidence, such as homeopathy^28^ or dietary supplements to treat dementia^29^, among many others. Previous research links the belief in pseudomedicines to a more general cognitive bias known as “causal illusion”, which implies the belief that one event causes another when, in reality, it does not^30^. What Vinas et al.^27^ found is that participants with abundant resources tended to administer the medicine to as many patients as possible, and thus, they tended to associate the pseudomedicine with the spontaneous remissions of the disease that eventually occurred, leading to an illusion that the pseudomedicine was working. By contrast, because participants with budget constraints reduced the use of pseudomedicine, they were more exposed to cases in which remissions occurred in the absence of the treatment, and thus, they showed a reduction in their causal illusion.

The results from Vinas et al.^27^ indicate, thus, that scarcity can influence the amount of treatment allocated to patients and, consequently, affect the judgments of treatment effectiveness. In the particular case studied by Vinas et al.^27^, the scarcity manipulation had a beneficial effect because, in the absence of budget limitations, the general tendency is to use the treatment much more often, which leads to causal illusions when the treatment is not actually working^31^. Thus, participants with scarce resources in that study tended to use the treatment more sparsely and this prevented the illusion. However, scarcity may not be beneficial to improve judgments in all conditions. In particular, we argue that it could be negative when it is combined with the assumption that certain types of patients can benefit from the treatment to a greater extent, as we have seen during the COVID-19 pandemic, where under scarcity conditions, certain patients were discriminated against (e.g., for reasons related to age or health status). Thus, the combination of scarcity conditions and a PCS should produce a rigid pattern of resource allocation, reserving the treatment for those patients who should presumably be prioritized. Then, from the literature on contingency learning, we know that the unbalanced exposure to treated vs. not treated patients that results from this allocation can affect the way people perceive the effectiveness of the treatment.

Following this rationale, the current experimental series aimed to model human decisions in a situation characterized by the coexistence of budget constraints and an erroneous PCS comprising two groups of (fictitious) patients. These groups were described as having different sensitivity to the treatment (although, in reality, the classification was wrong as both groups were identically sensitive). In principle, participants should be able to learn from the information presented in the individual trials (i.e., whether the individual patients recover or not when they take the treatment). However, it is possible that they trust the PCS more, so we can assess the potential bias induced by an incorrect classification system. We chose treatment sensitivity to create the patient groups because it is a variable often considered in the real-world clinical setting for personalizing cancer therapies, biomarker research, and drug design^3^. Additionally, it was our goal to provide a mistaken classification that could then be confronted with disconfirming evidence during the experiment, to test which had more impact on how the participants perceived the effectiveness of the treatment: patient classification or direct experience. Thus, a classification based on expected sensitivity to the treatment seemed adequate for our purposes.

In our experiments, we asked participants to imagine that they were doctors who had to decide whether or not to administer a treatment to a series of patients so that we could test the participants´ choices for both groups of patients. In addition to these choices (on who should receive a treatment), we also measured their judgments of treatment effectiveness, because they reflect how people predict the outcomes that will follow their choices^32^. This was conducted in two situations: when the treatment was effective (Experiment 1) and when it was not, (i.e. when it was a pseudomedicine; Experiment 2). First, we predicted that, in both experiments, participants in the group with scarce budget would administer the treatment less often to the (fictitious) patients classified as less sensitive. This would result in lower effectiveness judgments compared to participants in the group with abundant budget. Secondly, we predicted that participants in the group with abundant budget would distribute the treatment equally across both categories of patients, reducing the potential unfair allocation of treatment, and, therefore, leading to similar effectiveness judgments for the two patient groups. That is, we expected that the incorrect recommendation made by the PCS would be overridden by actual evidence only in the group that had access to abundant doses to treat the patients. By contrast, those with a limited budget would tend to differentiate between patient groups when allocating the drugs and subsequently perceive the treatment as more effective in one group than in the other.

## Experiment 1

### Method

#### Ethics statement

The Ethical Review Board of the University of Deusto approved the procedure of these experiments. All experiments were performed in accordance with the approved guidelines. Informed consent was obtained from all participants. We did not collect any personal or identifiable data.

#### Participants

We recruited 100 anonymous participants via the survey platform Prolific Academic^33^. We limited the sample to people speaking fluent English and who had not participated in previous experiments conducted by our research group. Due to unknown technical reasons, data from one participant was not recorded, so the final sample size was *N* = 99 (distribution by gender: 58 women, 37 men, and four people self-defined as other; age: *M* = 27.8, *SD* = 7.8). Participants were randomly assigned to one of two groups: scarce (*n* = 47) and wealthy (*n* = 52). The sample size was decided for practical/ethical reasons, with the intention to recruit as many participants as possible. A sensitivity analysis conducted before data collection indicated that with 100 participants, we could detect a small effect size, *f* = 0.14, with 80% power in the 2 × 2 interaction. We estimated 10 min for completing the experiment and paid the participants £1 for their time. The study was pre-registered (see https://aspredicted.org/hc6ju.pdf ). We did not define any exclusion criteria.

#### Procedure

The experimental task was an adaptation of the standard causal learning task (see^31^, also used in the previously described experimental series on the effects of scarcity on causal judgments^27^. We had two groups differing in the available budget to buy drugs: scarce and wealthy. Additionally, in the instructions, participants were told that there were two different categories of patients, classified as a function of their presumed sensitivity to the treatment as either highly-sensitive (marked with a ‘+’ symbol) or barely-sensitive (marked with a ‘-’ symbol). That is, the patient category variable was manipulated within-subjects.

At the beginning of the experiment, participants read the instructions. They had to imagine that they were doctors treating a rare (fictitious) disease with a (fictitious) drug. We asked them to heal as many patients as possible, and, at the same time, to manage the budget efficiently. That is, we used “naturalistic instructions”, because they presumably reflect what people usually do in real life. Indeed, this seems to be the default strategy for most participants (thus, removing the naturalistic component would probably have little impact), but highlighting this goal typically leads to stronger causal illusions^34^. Nonetheless, we also advised participants that this treatment was still under development, so its effectiveness had not yet been proven. We also informed that patients were classified according to their expected sensibility to the treatment. At the end, we told them either that the budget available to buy the drugs was very large and that “there was usually a surplus” (wealthy group), or that it was very tight and “usually run out” (scarce group).

**Fig. 1:**
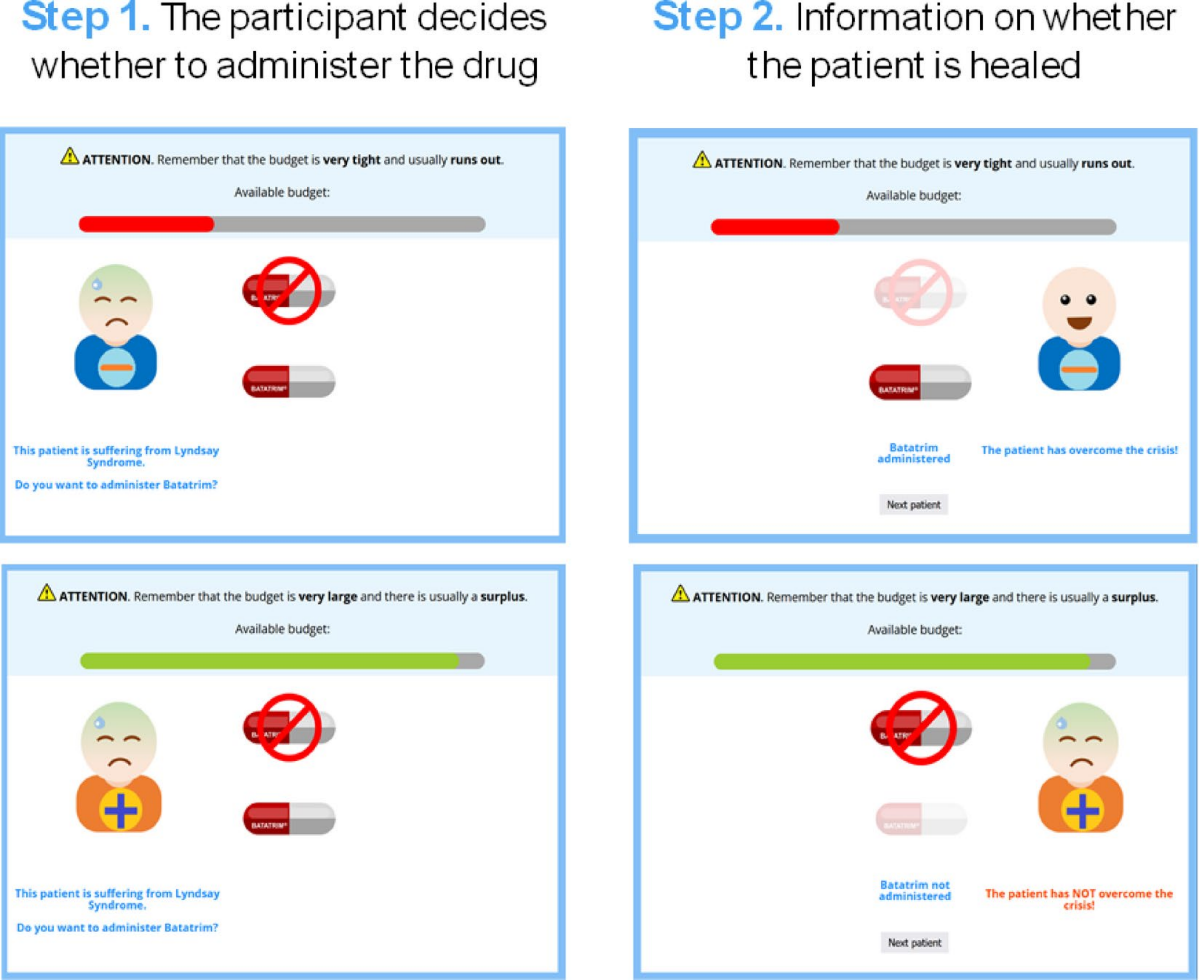
Screenshots showing examples of the two consecutive screens (steps) within each trial. The top panels refer to the scarce group, while the bottom panels refer to the wealthy group. Note how the budget bar and the reminder at the top of the screen indicate the different availability of resources for each group. The left panels show examples of the first screen, in which participants decide whether to administer the drug. The right panels show examples of the second screen, informing participants whether the patient has been healed. Recall that the probability of healing is 0.70 when the drug is administered and 0.20 when it is not.

After reading the instructions, participants proceeded to the learning phase, in which they visited a sequence of 60 patients in total, 30 classified as highly sensitive and 30 classified as barely-sensitive, in random order. Each trial portrayed an individual patient, and the participant had to choose whether or not to administer the drug by clicking on the corresponding button. Immediately afterward, the screen informed about the outcome by indicating whether the patient healed or not (see Fig. 1). Unbeknownst to the participants and independently of the patient category, 70% of the patients healed when they received the drug, and only 20% of them healed when they did not. This means that the drug was an effective treatment, but more importantly, it was identically effective for both categories of patients. That is, the categorization of patients was incorrect.

Our first variable of interest is P(C), or the probability with which the participants used the drug on each category of patients. To calculate this value, we took the number of doses given to each of the two patient categories and divided it by the total number of patients in the category (i.e., 30). This was our measure of the tendency to use the drug with each category of patient and it could take values between 0 and 1.

Additionally, to reinforce the budget manipulation, participants constantly saw a reminder of the available budget (scarce or abundant) and a budget bar (see Fig. 1). Each time they used one dose of the drug, the budget bar was updated by showing a reduction. This reduction was larger in the scarce group, where each dose administered reduced the bar by 1/60, as compared to the wealthy group, in which it reduced the bar by 1/600. We also used colors to reinforce the reduction: if the budget bar decreased to 1/2 or 1/3 of the initial budget, the color of the budget bar changed from the original green to orange, and then to red, respectively (note that, since the wealthy group had a large number of doses available, their bar was always green). Importantly, both groups had enough resources to buy doses for all their patients.

Once the participants had visited all 60 patients, we measured their judgment of the effectiveness of the drug for each patient category. This was our second dependent variable. We used a scale from 0 (completely ineffective) to 50 (moderately effective) to 100 (completely effective). In this experiment, the correct answer was 50, because the real effectiveness of the drug can be computed as the difference between the probability of healing when using the drug (70%) minus the probability of healing when not using the drug (20%). (Although small deviations from the programmed contingency are possible, we programmed the experiment to ensure that the total frequencies of each trial type were very similar to the actual frequencies. Nevertheless, we have also checked it. This analysis shows that the actual experienced contingencies did not depart much from the programmed ones (i.e., 0.5 in Experiment 1 and zero in Experiment 2). These data are reported in the Supplementary Analyses online file (including Tables S1 & S2). Finally, we presented the two effectiveness questions (one for each category of patients) on the same screen and randomized their position (top/down) for each participant.

### Results and discussion

#### Influence of the budget and patient category on P(C)

**Fig. 2:**
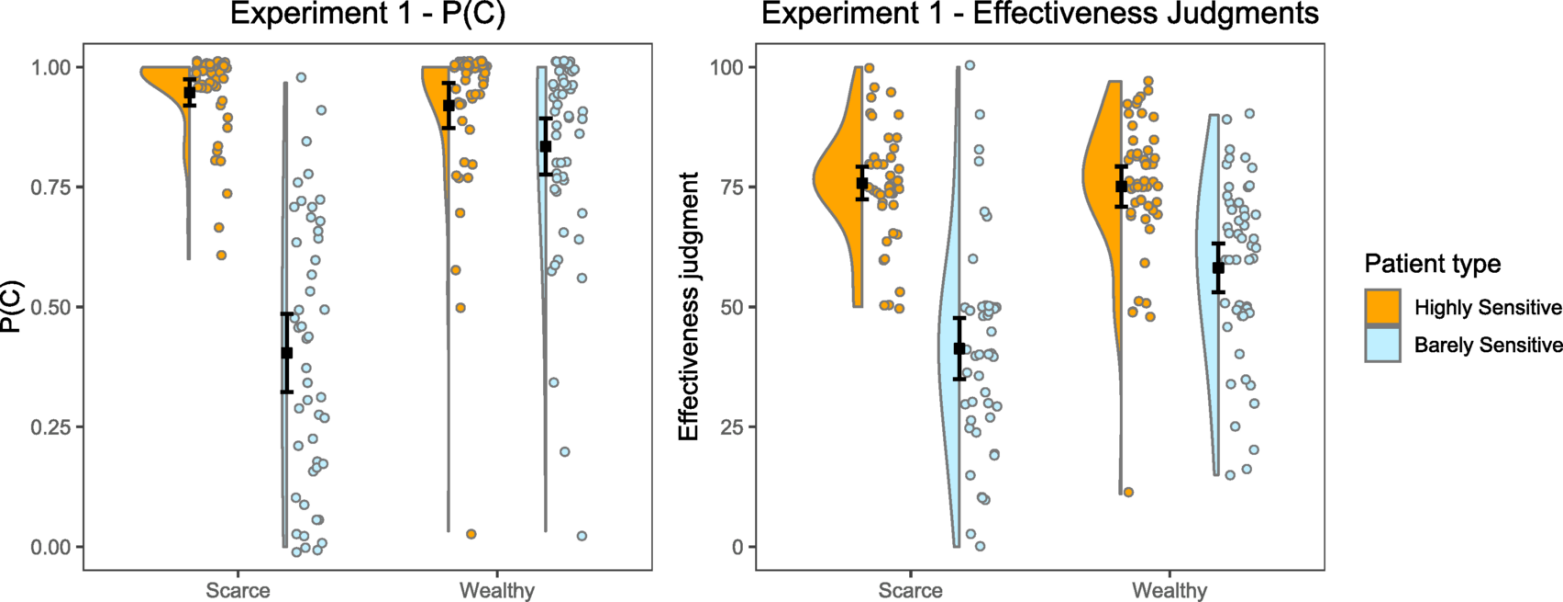
Experiment 1. Mean P(C) and Mean Judgment of Effectiveness as a Function of Budget and Patient Classification. Error bars depict 95% CIs for the mean.

Figure 2 shows the mean scores obtained on the two dependent variables, P(C) and judgment of effectiveness, in the two budget groups (scarce, wealthy) and the two intragroup conditions (highly-sensitive patients, barely-sensitive patients) in Experiment 1. We performed a 2 × 2 Mixed ANOVA with the factors budget (scarce, wealthy) and patient category (highly-sensitive, barely-sensitive) on the P(C). The results showed a significant main effect of budget, *F*(1, 97) = 55.0, *p* < .001, and a significant main effect of patient category, *F*(1, 97) = 115.5, *p* < .001. The interaction between budget and patient category was also significant, *F*(1, 97) = 61.3, *p* < .001. Subsequently, we examined the interaction through post hoc contrasts applying Tukey’s correction. First, the P(C) was significantly different between both budget groups for barely-sensitive patients, *t*(97) = 8.75, *p*_*tukey*_ < 0.001, so participants with scarcity administered the drug less often than those with abundance to those patients. Second, the P(C) was not significantly different between participants in both budget groups for patients classified as highly-sensitive, *t*(97) = 0.976, *p*_*tukey*_ = 0.763. Third, as Fig. 2 also shows, the P(C) was significantly different in the case of participants with scarcity between both categories of patients, *t*(97) = 12.81, *p*_*tukey*_ < 0.001. However, it was similar for participants in the group with abundance between both categories of patients, *t*(97) = 2.12, *p*_*tukey*_ = 0.155. In other words, the participants in the group with scarcity behaved as if the budget was abundant in the case of patients who had been classified as highly-sensitive, administering a high amount of medicine, while reducing it in the case of patients classified as barely-sensitive. On the other hand, participants with abundance administered similar amounts of medicine regardless of the category of patient. Additional analyses showing the evolution of the P(C) over the training trials per group and patient category are reported in the Supplementary Analyses online file (including its Figure S1).

#### Influence of the budget and patient category on the judgment of effectiveness

We also performed a 2 × 2 Mixed ANOVA with factors budget (scarce, wealthy) and patient category (highly-sensitive, barely-sensitive) on the judgment of effectiveness (Fig. 2). As in the case of P(C), the results revealed a significant main effect of budget, *F*(1, 97) = 10.9, *p* = .001, a significant main effect of patient category, *F*(1, 97) = 13.4, *p* < .001, and a significant interaction between budget and patient category, *F*(1, 97) = 13.4, *p* < .001. The interaction was examined through post hoc contrasts applying Tukey’s correction. First, we found significant differences for barely-sensitive patients depending on the budget, *t*(97) = 4.20, *p*_*tukey*_ < 0.001, so that participants in the scarce group produced lower judgments compared to those in the wealthy group, as Fig. 2 shows. Second, the participants with scarce and abundant budgets showed similar judgments for patients classified as highly-sensitive (see Fig. 2), *t*(97) = 0.26, *p*_*tukey*_ = 0.994. These judgments were, on average, well above the actual effectiveness of the drug (i.e., 0.50). Third, when comparing the patient categories against each other, we found that participants gave higher judgments for patients classified as highly-sensitive both when the budget was scarce, *t*(97) = 9.926, *p*_*tukey*_ < 0.001, and when it was abundant, *t*(97) = 5.12, *p*_*tukey*_ < 0.001. This latter result can be interpreted as participants basing their judgment of effectiveness not only on their experience during the experiment, but also, and perhaps to a greater extent, on the information they were given in the initial instructions, which indicated that according to the PCS the two groups of patients had different sensitivity to the drug.

Finally, once we had documented that budget manipulations can affect the judgments of effectiveness, we wondered whether we could explain this effect by how much participants used the drug during the training phase, as reported by Vinas et al. (2023). Thus, to test whether the P(C) mediated the influence of the budget on the judgment of effectiveness, we ran a mediational analysis for each patient category. In the models, budget group was the independent variable, effectiveness judgments were the dependent variables, and P(C) was the mediator. Considering patients classified as barely-sensitive, and as we expected, the P(C) completely mediated the total effect of the budget on judgments (β= -0.393, *Z* = -4.227, *p* < .001), as the indirect effect was significant (β = 0.682, *Z* = 6.626, *p* < .001) but the remaining direct effect was not (β = 0.060, *Z* = 0.583, *p* = .560). This means that, as in our previous studies, when participants chose to administer fewer doses, their judgments became smaller as a consequence. In contrast, in the case of patients classified as highly-sensitive, and consistent with the ANOVA results discussed above, we did not find that the total effect was significant, β = 0.027, *Z* = 0.263, *p* = .792. Thus, it was pointless to seek mediation because, for these patients, effectiveness judgments were not influenced by the budget.

In brief, we found that, in line with previous reports^27^, participants’ usage of a fictitious drug was modulated by the available budget. We also found that it was influenced by a PCS that classified patients according to their expected sensitivity to the treatment, even though this classification was incorrect and the participants had the opportunity to learn it. Moreover, faced with patients classified as barely-sensitive, the high or low usage of the drug served to modulate the participants’ judgments about the effectiveness of the drug, although the directly available information showed that the medicine was equally effective for all.

## Experiment 2

In Experiment 1, we examined the combined effect of providing a PCS and a budget manipulation on two variables: the probability of using a drug and the judgments of drug effectiveness. Participants were presented with a scenario in which the treatment was genuinely effective, reflecting real-life situations such as resource allocation decisions during crises like the COVID-19 pandemic (e.g., determining which patients receive scarce resources like antigen tests, vaccines, or respirators). The previous study, thus, illustrates this using a scenario in which a valid medical treatment is scarce. Importantly, in real life, there are not only effective treatments, but also ineffective treatments that people sometimes use as well. They are called pseudomedicines, and they consist of treatments whose effectiveness is not supported by scientific evidence^28^. In those cases, users can even develop the illusion that the treatments are effective, as several studies have already shown^35–38^. In times of scarce resources, such as during the COVID-19 pandemic, pseudoscience proliferates even in high income countries^39^. Indeed, and as has already been documented, the pandemic is an excellent example of how fraudulent products may flood the market when there is a scarcity of treatments^40^.

Therefore, it was also necessary to investigate the effects of PCS and scarcity in situations in which the treatments are ineffective. This was the aim of this second experiment. We used the standard causal learning task from Experiment 1 in a scenario in which using the treatment does not increase the probability of healing compared to when it is not used. Previous research on ineffective treatments indicates that people tend to overestimate their effectiveness^41^, and develop, therefore, what is called a causal illusion^36,37^. That is, people can overestimate the effectiveness of a treatment so that they end up believing that a completely ineffective treatment is working. Interestingly, scarcity has been identified as a variable that can reduce the causal illusion^27^, as it helps to keep a balance between using and not using the treatment, thus providing more comprehensive information than when people tend to administer the treatment as often as possible (i.e. when abundance exists). Hence, the aim of Experiment 2 was to extend the results of Experiment 1 (which involves a PCS and a scarcity manipulation) to a treatment that did not work. This is helpful for understanding the mechanics of believing in the effectiveness of pseudomedicines, when used in complex medical contexts, in which PCS and scarcity often co-exist.

### Method

#### Participants

One hundred ninety-one Psychology students participated in the experiment. They received course credit for participating, regardless of whether they opted to submit their data, which they decided by clicking either “Send data” or “Do not send data” at the end of the session. Twenty-one students chose not to submit their data, resulting in a final sample size of *N* = 170. This comprised 142 women and 28 men, (age: *M* = 18.6, *SD* = 0.87), randomly allocated to one of two groups (scarce group: *n* = 91, wealthy group, *n* = 79). Our sensitivity analysis indicated that with 170 participants we could detect a small effect size in the 2 × 2 interaction, *f* = 0.09, with 80% power. The study was pre-registered (see https://aspredicted.org/nn796.pdf ) and no exclusion criteria were used.

#### Procedure

The experimental task was similar to that of Experiment 1, with three changes:

(1) First, the most important modification was that, in this case, 70% of patients healed when they received the treatment, but the same proportion of patients (70%) healed when they did not receive it. In other words, the treatment was not an effective drug but rather a pseudomedicine because it was unable to increase the probability of healing as compared to when it was not received. Consequently, in this experiment, the correct judgment of effectiveness was zero (see Note 1). We chose a high percentage of healings because it is a situation that promotes the overestimation of treatment effectiveness^42,43^. (2) In addition, we introduced a minor change in the denomination of the two patient categories, from “highly” vs. “barely” sensitive (in the previous experiment) to a simpler dichotomous one, “sensitive” vs. “non-sensitive”. With this change, we expected participants to understand the patient categories more easily. (3) We conducted this experiment on a sample of Psychology students (instead of Internet users from Prolific Academic, as in the previous experiment). This change was due to practical reasons (namely, availability of resources to conduct the study), but in line with our previous record of replications with online and offline samples using this type of experimental task (see e.g^44,45^, we would expect no big differences depending on whether the study was conducted online or offline.

### Results and discussion

#### Influence of the budget and patient category on P(C)

**Fig. 3:**
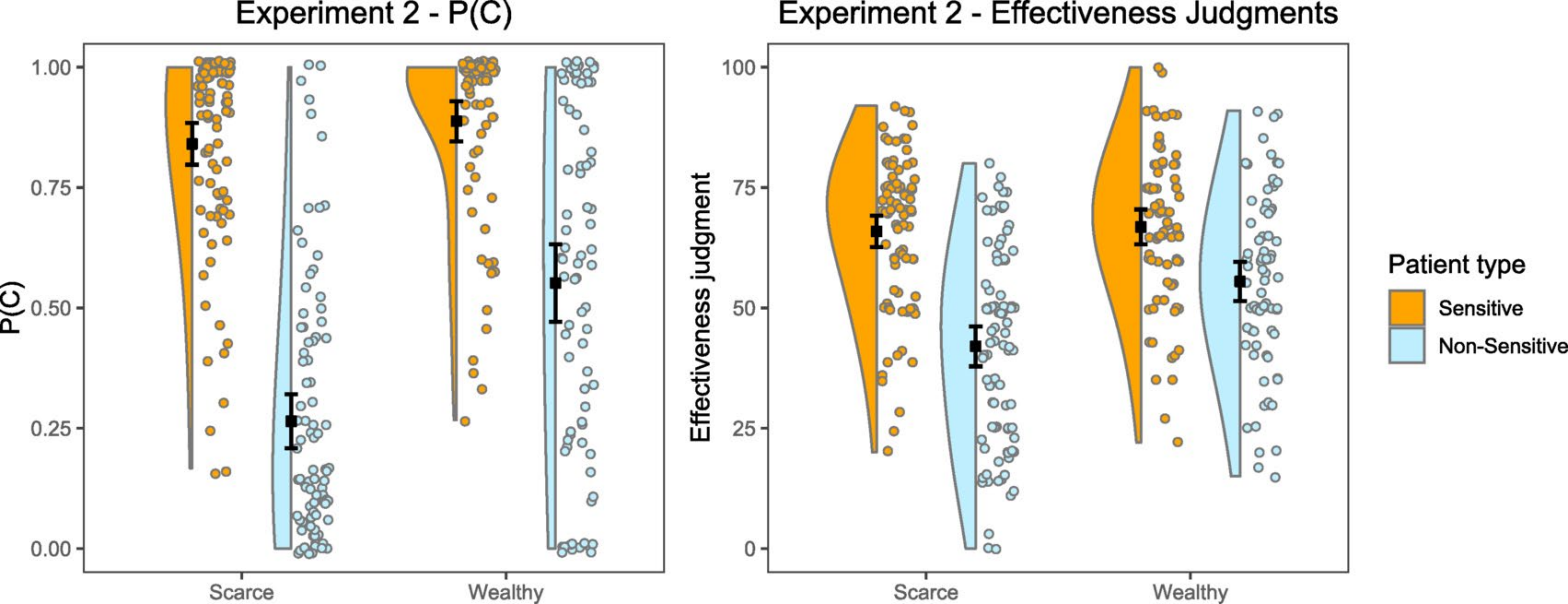
Experiment 2. Mean P(C) and Mean Judgment of Effectiveness as a Function of Budget and Patient Classification. Error bars depict 95% CIs for the mean.

Figure 3 depicts the P(C) for sensitive and non-sensitive patients in both budget groups. We conducted a 2 × 2 Mixed ANOVA with budget (scarce, wealthy) and patient category (sensitive, non-sensitive) as factors and P(C) as the dependent variable. The results showed that there was a main effect of budget, *F*(1, 168) = 53.8, *p* < .001, and a main effect of patient category, *F*(1, 168) = 186.0, *p* < .001. The interaction between budget and patient category was also significant, *F*(1, 168) = 12.9, *p* < .001. To break down the interaction, we compared the participants’ P(C) through post hoc contrasts applying Tukey’s correction. First, the P(C) was significantly different between participants in both budget groups for patients classified as non-sensitive, *t*(168) = 5.93, *p*_*tukey*_ < 0.001, so that participants with scarcity administered fewer doses to these patients compared to participants with abundance. Second, the P(C) was not significantly different between participants in both budget groups for patients classified as sensitive, *t*(168) = 1.55, *p*_*tukey*_ = 0.412, that is, participants administered similar amounts of pseudomedicine to these patients independently of their budget. Third, there were also significant differences in the P(C) for patients classified as sensitive and non-sensitive in both budget groups, with less pseudomedicine administered to the second ones as compared to the first ones: *t*(168) = 12.64, *p*_*tukey*_ < 0.001 in the case of participants with scarcity; and *t*(168) = 6.87, *p*_*tukey*_ < 0.001 in the case of participants with abundance. That is, as it happened in Experiment 1, when faced with patients classified as sensitive, the participants in the group with scarcity behaved as if the budget was abundant. However, unlike Experiment 1, this time participants reduced the amount of pseudomedicine administered to patients that were classified as non-sensitive in both budget groups (although participants with scarcity did it to a greater extent). Additional analyses showing the evolution of the P(C) over the training trials per group and patient category are reported in the Supplementary Analyses online file (including its Figure S2).

#### Influence of the budget and patient category on the judgment of effectiveness

We conducted a 2 × 2 Mixed ANOVA with budget (scarce, wealthy) and patient category (sensitive, non-sensitive) as factors on the judgment of effectiveness (Fig. 3). The results were similar to those concerning the P(C). Both the main effect of budget, *F*(1, 168) = 15.2, *p* < .001; and patient category, *F*(1, 168) = 78.5, *p* < .001, were significant, as well as the interaction between budget and patient category, *F*(1, 168) = 10.0, *p* = .002. We examined the interaction with post hoc contrasts applying Tukey’s correction. First, as in the case of the P(C), participants in the scarce group presented a significantly lower effectiveness judgment than the participants in the group with abundance for patients classified as non-sensitive, *t*(168) = 4.58, *p*_*tukey*_ < 0.001. Second, as Fig. 3 shows, participants presented a similar effectiveness judgment for patients classified as sensitive, independently of their budget, *t*(168) = 0.371, *p*_*tukey*_ = 0.983. These judgments were higher than the programmed effectiveness (recall that the correct effectiveness value was zero in all cases). Third, unlike the results in Experiment 1, in this case, the judgments of effectiveness behaved similarly to the P(C): there were significant differences between the judgments for patients classified as sensitive and non-sensitive in both budget groups, so the judgments of effectiveness were lower for non-sensitive patients compared to those for sensitive patients: *t*(168) = 8.82, *p*_*tukey*_ < 0.001 in the case of participants with scarcity, and *t*(168) = 3.89, *p*_*tukey*_ < 0.001 in the case of participants with abundance.

Next, we also tested the mediational model proposing that the P(C) mediated the effect of the budget on judgments. This model was conducted on each patient category separately. In the case of patients classified as non-sensitive, the results confirmed that the total effect of the budget on effectiveness judgment was significant, β = 0.333, *Z* = 4.59, *p* < .001. The mediation was total, since the indirect effect of the budget on effectiveness judgment through P(C) was significant (β = 0.207, *Z* = 4.57, *p* < .001), whereas the direct effect was not (β = 0.126, *Z* = 1.81, *p* = .070). In contrast, in the case of patients classified as sensitive, the total effect of the budget on the judgments of effectiveness was not significant, β = 0.372, *Z* = 0.37, *p* = .710. That is, given that there was no budget effect for patients classified as sensitive, there is no point in examining the mediation through P(C).

## General discussion

As noted in the Introduction, two factors that can affect medical decisions are the presence of patient classification systems and resource constraints. We hypothesized that these two factors, together, could produce or intensify existing biases in the context of medical decision-making, as we have illustrated with an example from the COVID-19 pandemic. In the current research, we designed a procedure in which participants acted as if they were doctors and we introduced these two manipulations to study how they affected (1) the participants´ choice to use a (fictitious) treatment on each patient of the different categories, and (2) their judgments about the treatment effectiveness. Moreover, we simulated two scenarios: one using medicine that worked to improve patients’ health (Experiment 1) and another using pseudomedicine that produced no effect (Experiment 2).

As already mentioned, our goal was to investigate whether the participants in our experiments would rely on their preconceptions about patient classification, as induced by the PCS, and whether scarcity (or abundance) would influence their decisions to administer the treatment and their judgments of effectiveness. All in a situation in which participants could get the information they needed to reach the correct conclusions by just looking at the effects of their actions. We expected that participants facing scarcity would give fewer doses to patients classified as less sensitive to the treatment. This would, consequently, reduce their judgments of effectiveness for these patients compared to the other category (although the real effectiveness was identical for both categories of patients). Conversely, we expected a different pattern in participants with an abundant budget: because they had enough resources, we expected that they would allocate the resources more uniformly, and, therefore, they could learn that the drug was equally effective regardless of the presumed patient category.

As expected, we found that patient classification and budget availability influenced the number of administered doses of both the effective drug (Experiment 1) and the pseudomedicine (Experiment 2), which in turn affected the judgments about their effectiveness.

We also found that different categories of patients (erroneously classified) received similar amounts of the drug when the budget was abundant. Probably this was because participants learned that the treatment was similarly effective from the experience they acquired during the training phase and to some extent disregarded the instructions of the patient classification. Surprisingly, however, the effectiveness judgments of the wealthy participants were also lower in the case of patients classified as less sensitive. These judgments seem to reflect the information the participants received from the PCS at the beginning of the experiment, regardless of what they had then learned throughout the learning phase. That is, participants considered the PCS information over and above their own experience when judging the effectiveness of the treatment.

In Experiment 2, when confronted with a drug that was ineffective (i.e., pseudomedicine), participants with scarcity used it as if they were wealthy, only on patients classified as more sensitive. Conversely, possibly due to concerns about budget limitations, these participants adopted a strategy of efficiency, significantly reducing the use of the drug on patients classified as less sensitive. Therefore, the effectiveness judgments of participants were also different as a function of the patient category. Thus, participants developed a significantly higher effectiveness illusion for patients classified as more sensitive. Notably, this difference between patient types was especially pronounced in participants facing scarcity. That is, participants chose to administer the drug preferentially to those patients they expected to get the most gain of it. This is consistent with the thesis of^38^, who state that the main reason why people use pseudomedicines and pseudotherapies is the belief that they work.

The results of these two studies are robust and have practical consequences. Faced with an effective drug (Experiment 1), the main error of the participants was, as previously mentioned, to rely more on the established classification than on the learning phase. This was particularly clear in participants whose budget was scarce, because they decided to use the drug less often on patients classified as less sensitive, and this in turn led them to receive incomplete data during the learning phase, and thus to reach lower judgments of effectiveness for the effective drug. This is important because in real life, health professionals usually base their practice on patient classification systems, and these classifications can be wrong for many reasons. For example, because they are not always based on objective data^9–11^, or just because they are outdated or incomplete. As a consequence, in the face of effective treatments, and especially when scarcity exists, a wrong classification of patients can result in unfair allocation of healthcare resources. Even when the actual experience acquired from the observation of the different cases during the learning phase suggests that the classification was wrong, this classification can also result in biased judgments of effectiveness, and therefore, in biased future predictions^32^.

On the other hand, in the face of a pseudomedicine (Experiment 2), those errors also occurred, but an additional one was to incorrectly judge that the treatment was effective when it had no effect (i.e., a causal illusion). As previous literature has shown, the development of causal illusions is common when the use of the potential treatment and the healings are frequent. That is, when there are many coincidences between the potential cause and its presumed effect, even if they happen by chance^37^, such as, for instance in cases of self-limited diseases^41^. Particularly in conditions of abundance, people will tend to administer the drug more often and therefore the risk of developing an illusion of effectiveness will be high. By the same reasoning, particularly in conditions of scarcity, the belief that a patient possesses characteristics that render them more sensitive to the drug can be a risk factor that increases both the use of pseudomedicines and the erroneous judgment of effectiveness, with the resulting consequences: economic and time costs, but above all, a health risk due to the cost of opportunity as the pseudomedicine replaces a valid treatment^35^. We think it is especially relevant to consider this problem in emergencies, where scarcity of resources, time, and information about patients might become risk factors leading to bad decisions.

One potential limitation of our current research is that it is based on laboratory experiments. Laboratory studies are in fact useful to study scarcity, because they allow us to experimentally manipulate it without affecting the real resources of the participants. However, the generalizability of the conclusions can be, to some extent, compromised. This is why we agree with other researchers (for example,^23^ that it would be interesting to extend this research to real-life situations, where actual resources are scarce. The second limitation is that, during the experimental task, our participants solved a problem that was arguably different from the type of judgments and decisions they typically make about medical treatments in their real life (e.g., whether to take them themselves or administer them to a family member). Rather, they were asked to imagine a fictitious scenario in which they played the role of doctors. Although this may limit the generalizability of the results, we chose to use this task because it is adapted from the standard causal learning task (see^37^, which facilitates the comparability of our results with previous studies.

Additionally, and more importantly, we did not run the experiments with medical professionals (rather, our sample consisted of Internet users in Experiment 1, and Psychology students in Experiment 2), so there could be concerns about the applicability of our findings to fields such as medical practice. We decided to sample non-professional participants because previous research indicates that the cognitive biases of clinicians are similar to those of the general population^46,47^, and because it avoids the influence of factors like the degree of experience or the type of clinical specialty. Additionally, in Experiment 2 we used a sample of students of the degree in Psychology, which in Spain is legally considered as a health/clinical profession and therefore it could be understood as an adjacent population to clinicians, doctors and healthcare givers. Nevertheless, we think it would be important to replicate our research on a sample of healthcare professionals to see if, as expected, the findings hold.

Related to the previous point, we also point out that the two experiments differ in a number of attributes: not only the samples were obtained from different sources (Internet users vs. Psychology students), but they also varied in their gender and age distribution, the language used (Spanish vs. English), and in the specific wording of the patient labels. We do not have reasons to believe that these changes in the demographics and other details would affect the results systematically: there is no evidence, to our knowledge, of variables like age and gender affecting the performance in this type of task systematically, and we have documented that online and offline samples reproduce the same effects in this experimental task^44,45^. Concerning the language chance, we note that all participants in both experiments did the study in their native language. However, we cannot discard that these factors play a role and thus we acknowledge that it is a limitation for comparing between the two studies. Note that within-experiment conclusions are unaffected, though.

Finally, our experiments explore the effect of resource availability in combination with an incorrect classification of patients, and we did not study effective vs. non-effective treatments within the same experiment. Thus, we did not conduct a single study with the complete design with all factors crossed, in which a PCS could be either right or wrong, resources could be either abundant or limited, and effectiveness could be either high or null. This is stated as a limitation, because we cannot draw conclusions as to which factors are more important than others or how they interact when combined. More importantly, we have not studied the case of a correct PCS that can be then confirmed during the task. These are questions that are open for future research. On the other hand, we aimed to keep the design simple, while still resembling some real-life situations with interesting consequences (as the examples described in the Introduction aim to illustrate concerning the COVID pandemic), and we believe that the current two experiments fulfilled this goal.

In sum, the current research shows how two common conditions in medical decision contexts, namely resource scarcity and the use of patient classifications systems potentially incorrect, could interact to increase unfair allocation of medical resources and reduce the accuracy of future predictions of treatment effectiveness. These predictions can be wrong in two ways: by erroneously assuming that different patients are differentially sensitive to the treatments, and by attributing effectiveness to a completely ineffective treatment.

## Electronic supplementary material

Below is the link to the electronic supplementary material.

Supplementary Material 1

## Data Availability

Data and materials for these experiments are openly available in the Open Science Framework at https://osf.io/yjzmr/.

## Code availability

Experiments were preregistered in https://aspredicted.org [Links for each experiment are shown at the method sections of each experiment].
