# Supplementary material for: The combined effect of patient classification systems and availability of resources can bias the judgments of treatment effectiveness
Source: Sci Rep. 2025 May 7;15:15915. doi: 10.1038/s41598-025-01043-w (PMC12059125; doi:10.1038/s41598-025-01043-w)
Supplement: Supplementary file 1 — Supplementary Material 1 [file 41598_2025_1043_MOESM1_ESM.docx]

**Supplementary Materials**

***The combined effect of patient classification systems and availability of resources can bias the judgments of treatment effectiveness.***

**Aranzazu Vinas, Fernando Blanco, and Helena Matute**

**Evolution of P(C) over the training trials**

***Experiment 1***

Figure S1 shows the evolution of the average P(C) per group and patient category over the training trials (grouped in 6 blocks of five trials). We ran a 2 x 2 x 6 mixed ANOVA (Budget [scarce, wealthy] x Patient category [highly sensitive, barely sensitive] x Block [1, 2, 3, 4, 5, 6]) on P(C). We found a main effect of block, *F*(5, 485) = 4.481, *p* < .001, and the interaction between block and patient category, *F*(5,485) = 5.485, *p* < .001, was significant. The interaction between block and budget, *F*(5, 485) = 0.409, *p* = .842, and the interaction Budget x Block x Patient category, *F*(5,485) = 0.945, *p* = .451, were non-significant.

Post hoc contrasts showed that in the case of patients classified as highly sensitive, there were no significant differences between blocks, i.e., P(C) was similar in all blocks regardless of budget (minimum *p_tukey_* = .955). Although the three-way interaction was not significant, we analyzed the P(C) on each group to better understand the pattern of results. Post hoc contrasts showed that, for patients classified as highly sensitive, there were no significant differences in the P(C) between blocks. This was found both for participants with abundant (minimum *p_tukey_* = .955) and scarce resources (minimum *p_tukey_* = 1). In the case of patients classified as barely sensitive, the same pattern was found only for participants with scarce budget (minimum p*_tukey_* = .699). However, the P(C) for barely sensitive patients in the abundant resources group showed a significant increase from block 1 to block 3, *t*(97) = -4.026, *p_tukey_* = .021. The rest of the contrasts between blocks were not significant. Taken together, the results suggest that overall there were no differences between trial blocks, except for barely sensitive patients, which increased slightly from the beginning of the session to block 3 (although the increase was only significant for the wealthy group). From block 3 onwards, P(C) seems to remain stable, suggesting that if had increased the training length, we would have observed similar results.

**Figure S1**

*Experiment 1. P(C) per Block of Trials in each Group and Patient Category*

*Note.* Error bars depict 95% CIs for the mean.

***Experiment 2***

Figure S2 shows the evolution of the average P(C) per group over the training trials (grouped in blocks of five trials). We ran a 2 x 2 x 6 mixed ANOVA (Budget [scarce, wealthy] x Patient category [sensitive, non-sensitive] x Block [1, 2, 3, 4, 5, 6]) on P(C). We found a main effect of block, *F*(5, 840) = 2.38, *p* = .037, an interaction between block and patient category, *F*(5, 840) = 4.78, *p* < .001 and an interaction Budget x Block x Patient category, *F*(5, 840) = 2.35, *p* = .04. The interaction between block and budget was non-significant, *F*(5, 840) = 1.90, *p* = .091.

**Figure S2**

*Experiment 2. P(C) per Block of Trials in each Group and Patient Category*

*Note.* Error bars depict 95% CIs for the mean.

Post hoc contrasts showed that in the case of patients classified as non-sensitive, there were no significant differences between blocks, i.e., P(C) was similar in all blocks. This was found for the wealthy group (minimum p*_tukey_ = .95*) as well as for the scarce group (minimum p*_tukey_ = .954*). In the case of patients classified as sensitive, we found non-significant differences between blocks in the wealthy group (minimum p*_tukey_ = .12*). However, we found three contrasts that were significant: the difference between the P(C) of block 1 and block 3, *t*(168) = 5.336, *p_tukey_* < .01; of block 1 and block 5, *F*(168) = 4.892, *p_tukey_* < .01; and of block 1 and block 6, *F*(168) = 5.638, *p_tukey_* < .01. There were no other significant differences from block 2 onwards. These results also seem to indicate that providing a longer training with more trials would not have led to a modification in the amount of treatment.

**Actual Frequencies of healings**

We examined the individual data and coded each trial type according the 2x2 matrix that is usually used for computing contingency: those patients who received the treatment and healed were coded as cells a, those who received the treatment but did not recover were coded as cells b, those who did not receive the treatment but recovered were coded as cells c, and those who did not receive the treatment and did not recover were coded as cells d. Then, we counted the occurrences of each cell to obtain the two conditional probabilities that convey the contingency information: the frequencies of cells a and b serve to compute P(Healing|Treatment) = a/(a+b), while the frequencies of cells c and d serve to compute P(Healing|NoTreatment) = c/(c+d). Table S1 shows these computed probabilities per group and patient category in Experiment 1. We can see how the deviations from the programmed values (0.70 and 0.20) are minimal.

**Table S1**

*Experiment 1. Mean Actual Frequencies (and Standard Deviation) per Group and Patient Category*

|  |  | Scarce  Highly sensitive |  | Scarce  Barely sensitive |  | Wealthy Highly sensitive |  | Wealthy Barely sensitive |
| --- | --- | --- | --- | --- | --- | --- | --- | --- |
| P (Healing \| Treatment) |  | 0.698 (0.011) |  | 0.624 (0.202) |  | 0.685 (0.099) |  | 0.674 (0.102) |
| P (Healing \| NoTreatment) |  | 0.225 (0.352) |  | 0.164 (0.071) |  | 0.204 (0.26) |  | 0.195 (0.164) |

Likewise, Table S2 shows the computed conditional probabilities, but for Experiment 2. In this case, the programmed values are 0.70 and 0.70, and deviations from these values are small.

**Table S2**

*Experiment 2. Mean Actual Frequencies (and Standard Deviation) per Group and Patient Category*

|  |  | Scarce Sensitive |  | Scarce Non-sensitive |  | Wealthy Sensitive |  | Wealthy Non-sensitive |
| --- | --- | --- | --- | --- | --- | --- | --- | --- |
| P (Healing \| Treatment) |  | 0.693 (0.052) |  | 0.675 (0.253) |  | 0.685 (0.046) |  | 0.689 (0.094) |
| P (Healing \| NoTreatment) |  | 0.703 (0.243) |  | 0.692 (0.081) |  | 0.656 (0.305) |  | 0.643 (0.201) |
